# Educational Robotics and Robot Creativity: An Interdisciplinary Dialogue

**DOI:** 10.3389/frobt.2021.662030

**Published:** 2021-06-16

**Authors:** Alla Gubenko, Christiane Kirsch, Jan Nicola Smilek, Todd Lubart, Claude Houssemand

**Affiliations:** ^1^ Departement of Education and Social Work, Institute for Lifelong Learning and Guidance, Luxembourg, Luxembourg; ^2^ Université de Paris et Université Gustave Eiffel, LaPEA, Boulogne-Billancourt, France

**Keywords:** creative robotics, human creativity, cognition, embodied creativity, educational robotics, human-robot collaboration, machine learning

## Abstract

There is a growing literature concerning robotics and creativity. Although some authors claim that robotics in classrooms may be a promising new tool to address the creativity crisis in school, we often face a lack of theoretical development of the concept of creativity and the mechanisms involved. In this article, we will first provide an overview of existing research using educational robotics to foster creativity. We show that in this line of work the exact mechanisms promoted by robotics activities are rarely discussed. We use a confluence model of creativity to account for the positive effect of designing and coding robots on students' creative output. We focus on the cognitive components of the process of constructing and programming robots within the context of existing models of creative cognition. We address as well the question of the role of meta-reasoning and emergent strategies in the creative process. Then, in the second part of the article, we discuss how the notion of creativity applies to robots themselves in terms of the creative processes that can be embodied in these artificial agents. Ultimately, we argue that considering how robots and humans deal with novelty and solve open-ended tasks could help us to understand better some aspects of the essence of creativity.

## Introduction

Enhancing the ability to generate unique and useful ideas in both humans and artificial agents is a crucial challenge for 21st-century problem solving. The ways in which humans and robots may engage in the creative process and foster the development of creative productivity is a central research question that interfaces psychology and technology. Robots have been a feature of modern culture since the early pulp fiction stories and Isaac Asimov’s literary contribution. Interestingly, Robbie the Robot was one of the stars of this early period, and finally became a featured “agent” in a 1956 classic science fiction film, entitled *Forbidden Planet*. Robby the Robot, who was human-sized, possessed artificial intelligence and was a problem solver who helped humans during space missions. More recently, Robby the Robot has re-appeared, in a miniature format, as a toy that children can learn to program. Although the idea of incorporating robots into our everyday lives might have seemed outlandish and flat-out unrealistic some decades ago, the presence of robotics has well expanded, even into classrooms.

The pedagogical motivation for connecting robots with pupils is the hypothesis that creativity may be fostered through human-machine interactive exchanges. The scientific literature highlights a number of experiments of this type which seem to produce positive effects on both children and machines. Thus, this article seeks to 1) exemplify through a synthesis of the literature what creativity-related aspects are covered by the field of educational robotics, 2) present the mechanisms underlying creativity which are potentially at work in these pedagogical situations and, thus, 3) understand better how children but also artificial agents can develop their creative expertize from physically and socially situated practices.

## A Short Overview of Educational Robotics

The term “educational robotics” refers to a field of study that aims to improve student’s learning experiences through the creation and implementation of activities, technologies, and artifacts related to robots (Angel-Fernandez and Vincze, 2018). In practice, these activities can involve the use of a physical robot, may that be a modular system like LEGO Mindstorms, or robots specifically constructed for the designated activities.

Such activities can be conceptualized for students from elementary to graduate levels and may include design, programming, application, or experimentation with robots. Educational robotics activities usually consist of the use of a robotics kit, with which children learn how to build and program the robots for a given task (Jung & Won, 2018). These activities can take the form of interventions, after-school activities, voluntary classes, or an entire course module focusing on robotics.

The theoretical foundations for the application of educational robots are multiple, but the constructionist educational approach has been the norm (Kafai and Resnick, 1996; Papert, 1981; Danahy et al., 2014). Robotics kits provide a modular approach regarding programming and building, often used as creativity-enhancing interventions in the school context. In working with these kits, students can exert engineering competencies and creative^1^ solutions to a vast array of problems, starting from making a robot move from point A to B. Furthermore, principles such as problem-based learning and gamification are guiding the implementation of educational robotics interventions. The latter, gamification, describes the use of game elements in non-game contexts to foster motivation (Sailer et al., 2014).

The robots’ humanoid appearance may foster student engagement (Zawieska et al., 2015). The characteristics of robotic devices themselves can yield interesting effects as well. In interviews with students who underwent a course including the use of robotics, Apiola et al. (2010) found that the playful aspect of robotics, partnered with the physical embodiment of learning contents, had an important role in students’ engagement. An exploratory qualitative study by Nemiro et al. (2017) emphasized the role of robotics in creating an engaging classroom atmosphere.

## Overview of Existing Interventions Using Robotics to Foster Creativity

An early theoretical stance on creativity in children was developed by Vygotsky (1967), who argued that creativity would develop out of playful activities in which children engage. During these play activities, not only past experiences would be engaged, but a sort of combinatory imagination would encompass newly formed impressions stemming from new realities. Guilford (1950) asked why schools do not engage more thoroughly in the fostering of students’ creative abilities.

In 1972, Papert and Solomon published “Twenty Things to Do with a Computer”, in which they proposed a further integration of Information and Communication Technology into school curricula. In the article, the authors presented a robot called “Turtle”, which is an early example of an educational robotics device (Papert and Solomon, 1972). This rather simplistic and non-anthropomorphic robot was directed to move around via an easy-to-learn programming language called “LOGO”. Papert and Solomon described how “Turtle” could be programmed to draw pictures on the surface on which it moved via a pen that was located on the center bottom of the robot.

In the early 2000s, robotic toolkits gained an ever-growing attention in the pedagogical context (Alimisis, 2013). Wang (2001) described the use of a robotics course for engineering students, stating that LEGO robotics would be “an excellent medium for teaching design, programming and creativity” (Wang, 2001, p. 5). However, this work focused mainly on promoting engineering education content and did not include a standardized creativity measure.

Adams et al. (2010) interviewed engineering undergraduates who completed a voluntary robotics module. Among other engineering problem-solving tasks, the module involved programming a LEGO Mindstorms robot. After this module, 64% of participants stated that their creative thinking skills had improved.

Cavas et al. (2012) investigated the effect of a LEGO Mindstorms robotics course on student’s scientific creativity. The sample consisted of 23 twelve-to thirteen-year-old students, attending a Turkish private school. During the course, the students were introduced to building and programming robots. The authors did not specify their measure of scientific creativity but stated that it increased in students after the program.

Álvarez and Larrañaga (2013) examined how a robotics intervention using LEGO Mindstorms affected student’s motivation and their improvement in algorithm coding abilities. Via short self-report questionnaires, the authors established an increase in the student’s motivation and course interest.

Huei (2014) implemented a five-week program in which freshmore students were introduced to a programming language for coding robots. After the program, 93.25% of the 74 participants agreed or strongly agreed that the mini-project had enhanced their creativity, research and problem-solving skills (Huei, 2014). Jagust et al. (2017) presented the results of workshops for gifted elementary students using LEGO Mindstorms robotic sets. Although the authors did not psychometrically assess creativity, their qualitative analysis concluded that the children were “creatively productive” (Jagust et al., 2017).

In the context of educational robotics, the term “programming” applies also to younger pupils, considering that simple, visual programming interfaces are widely available. Using these already available or self-designed robotics kits, students are often given a specific problem to solve. Sullivan and Bers (2018) provide an example of this kind of intervention; in their study, the children were asked to program a robot to move in accordance with a given dance. During the curriculum, the researchers used Positive Technological Development checklists for observing the pupil’s behavior during the intervention. Sullivan and Bers (2018) stated that the frequency of creative behavior observed during the curriculum was “relatively high” (Sullivan and Bers, 2018). Creative behavior was associated with the use of a variety of materials or with using affordances of the materials in unexpected ways.

In some studies, the effects of educational robotics on student’s creativity were examined using standardized creativity measures. Alves-Oliveira (2020) investigated whether scholastic activities with robots would enhance children’s creativity. Children’s creativity levels were assessed in three conditions. In the first condition, children performed STEAM activities by learning how to code robots. In the second condition, children performed these activities by learning how to design robots. The third, control, condition, was comprised of children engaging in a music class. The pretest-to-posttest evolution in creativity was assessed with the Test for Creative Thinking-Drawing Production–TCT-DP (Urban and Jellen, 1996). In the TCT-DP, the examinee must finalize an unfinished drawing, and several variables, including new elements added, are evaluated. Results showed that creativity levels were boosted after each intervention. When examining the change in overall creativity scores, associated with each condition, the coding condition yielded a larger effect size than the control and the design condition. The TCT-DP assesses two creativity dimensions, namely: adaptiveness and innovativeness (Lubart et al., 2010). The effect of the design intervention on children’s creativity was mainly explained by an increase in scores on the TCT-DP innovativeness dimension, which is related to unconventional ways of thinking. According to Alves-Oliveira (2020), this dimension is associated with divergent thinking.

Alves-Oliveira (2020) argued that the nature of the coding task, which involved learning via trial and error, stimulated non-conventional thinking in the children. More specifically, in the coding condition of this study, the children learned how to use “Scratch language” (Resnick et al., 2009 in Alves-Oliveira, 2020). The young participants were divided into groups of 3–4 participants. Each group was appointed to program a mail-delivery robot. The robot was directed by simple codes written by the pupils, which made the robot move from one place to another. According to Alves-Oliveira (2020), this fostered a strong effect of the coding condition on the “stimulation of non-conventional ways of thinking”. The author argued that the nature of the coding task explained the larger effect size on children’s “innovativeness”, observed in the coding condition; the children were forced to experiment and explore during the coding tasks and learned by trial and error. Alves-Oliveira (2020) concluded that this learning via trial and error stimulated non-conventional thinking.

Eteokleous et al. (2018) conducted a study in which 32 primary school students between 5 and 12-years old participated in a 1-h non-formal robotics curriculum once per week. In order to assess the effects of the curriculum on student’s creativity, the Torrance Test of Creative Thinking, TTCT (Torrance, 1974), was administered before and after the 36-week intervention. Comparisons of the creativity scores before and after the intervention indicated a significant improvement in children’s creative abilities (Eteokleous et al., 2018).

Badeleh (2019) examined the effects of a robotics construction course on 120 student’s creativity and physics learning. A constructivist robot learning approach was used, which means that the learning outcomes were mainly acquired through the construction and testing of a robot with the use of a prepared manual. Badeleh (2019) implemented a study design, which included an experimental and a control group. The control group received traditional physics classes. The Torrance Creativity Questionnaire (Torrance, 1974 as cited in; Badeleh, 2019), assessing the dimensions of fluidity, flexibility, innovation, and detailed explanation, was administered to both groups before and after the intervention. The results showed that the constructionist robotics training had significantly increased student’s global creativity.

Hendrik et al. (2020) examined whether the use of robotics as learning tools has a positive effect on Figural Creativity (FC) in 40 elementary school students. The educational robotics intervention consisted of seven weekly lessons of 2–3 h. After the first introductory lesson, students participated in robot designing projects. To assess possible changes in FC, Hendrik et al. (2020) used the Torrance Figural Creativity Test (Torrance, 1974) before and after the intervention. Hendrik et al. (2020) defined the purposes of each lesson beforehand, and which of the four dimensions (fluency, flexibility, originality, elaboration) of the Torrance Test would be targeted each time. In one lesson, students were asked to construct an anthropomorphic robot, using LEGO Mindstorms sets. According to Hendrik et al. (2020), an important outcome of this lesson was to raise the student’s attention to the fact that different types of robots (humanoid and non-humanoid) could be built with the same robotics kit. The pretest-to-posttest comparisons of global FC scores indicated that they had increased in the intervention group. Therefore, Hendrik et al. (2020) advocated the inclusion of robotics classes in school curricula.

To summarize, a substantial amount of work dedicated to Educational Robotics (ER) has been conducted. Although many studies on ER include the notion of “creativity”, they refer mainly to problem-solving abilities. At times, creative abilities were exclusively assessed with self-report measures. Other studies, which relied on standardized instruments, such as the TCT-DP or the TTCT, observed increases in participant’s *Innovativeness* (Alves-Oliveira, 2020), *Closure* and *Creative Strength* (Eteokleous et al., 2018). In general, studies that examined the effects of ER on creativity rarely made use of clearly defined creativity constructs, and often did not provide a detailed account of the revealed effects.

Future studies could explore the underlying cognitive aspects of ER interventions, with reference to standardized creativity measures. One line of work could investigate the specific impact of ER interventions on ideational fluency, flexibility, and originality. Another line of work could examine the differential effects of specific types of ER activities, such as differences between designing robots vs. programming robot kits for a specific task. In practice, that could result in an examination of cognitive outcomes related to either designing or programming robots. However, in order to understand the underlying cognitive processes of ER interventions, clearly defined, operationalized and transferable theoretical frameworks are necessary.

## Multivariate Approach to Creativity–Confluence Model

In the multivariate approach to creativity, the confluence model (Lubart et al., 2015) considers how cognitive, conative, affective, and environmental aspects synergistically interact with the requirements of a particular field to give birth to a creative product. Cognitive aspects refer to intelligence, knowledge, and information processing abilities. Conative aspects refer to personality traits and motivation. With regards to personality, perseverance, tolerance of ambiguity, openness to new experiences, and risk taking are particularly important for creativity. The creative process does not unfold in a vacuum, however. Environment plays an important role in the translation of creative potential into a creative product.

**FIGURE 1:**
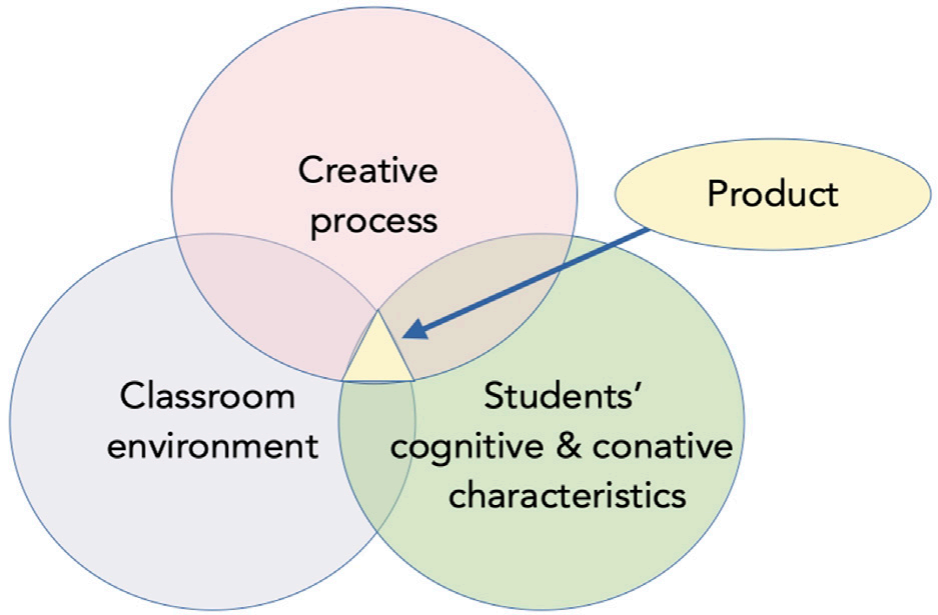
Confluence model for educational robotics. Note: This figure is adapted from Nemiro et al. (2017).

Educational robotics provides an excellent opportunity to study how real-world creativity emerges from student’s interaction with their social, physical, and cultural environment (Figure 1). In robotics activities, students learn to use affordances and constraints of robotic construction kits while engaging in collaborative problem solving in order to build their authentic and functional robotic device. These activities perfectly instantiate Glăveanu’s definition of creativity (Glăveanu, 2013, p.76), which is “the action of an actor or group of actors, in its constant interaction with multiple audiences and the affordances of the material world, leading to the generation of new and useful artifacts”.

While recognizing the role of conative factors, in this work, we will pay special attention to student’s cognitive processes and strategies because we suppose that non-cognitive factors act upon cognitive ones. In the following sections, we will consider creativity as situated practice and explain the positive effect of educational robotics on student’s cognitive mechanisms. However, before considering the mental process involved in robotics training, we will describe the creative process itself.

## Existing Models of Creative Cognition

One of the first models of creative thinking was proposed by Wallas (1926). His four-stage model comprised preparation (problem finding, problem analysis, and acquisition of domain skills and knowledge), incubation (putting the problem aside for a while without consciously thinking about it), illumination (a sudden burst of insight), and verification. Walla’s model not only emphasized the role of meta-components such as problem definition and evaluation but also stressed the role of uncontrolled, unconscious processing in idea generation. Although the model is intuitively appealing, it has been noted that not all creative solutions arise from a spontaneous “Aha”! or “Eureka” experience. The creative idea can also be a result of deliberate problem-solving efforts (Weisberg, 1986; Finke, 1996; Dietrich, 2004). As such, a comprehensive model should give a more detailed account of cognitive operations underlying the solution-finding process. Moreover, whereas the creative process is described as linear, the real-life creative problem solving is dynamic, has a loosely structured sequence, and does not necessarily follow a linear structure (Mumford et al., 1991; Schön, 1983; Corazza and Agnoli, 2018; Lubart, 2018). Despite these drawbacks, the Walla’s model (1926) has had an enormous impact on modern conceptions of the creative act and represents the first account of the creative process as involving explicit and implicit mechanisms.

Building on the model of Wallas, Amabile (1983) proposed to make a distinction between 1) the problem identification and 2) preparation stages. According to Amabile, during the former, problem definition and construction take place, whereas the latter is where reactivation of knowledge and search for task-relevant information happen. Amabile has also replaced a black-box illumination phase by 3) response generation phase and defined it as seeking and producing potential responses. She has suggested that the solution generation process represents a flexible (sometimes even random) search of possible pathways and exploring the environment’s characteristics. In other words, this stage involves searching for productive heuristics, which are defined as any principle or device that provides useful shortcuts for solving novel problems. Amabile argues that the choice of strategy (a set of heuristics) is crucial as it determines the level of novelty of the final solution. This idea draws upon the information-processing model of cognition by Newell and Simon (1972) and has received empirical support in creativity research (Spiridonov, 1997; Gilhooly et al., 2007; Nusbaum and Silvia, 2011). Newell and Simon hypothesized that people can solve unfamiliar problems because they can choose among alternative actions, anticipate the outcomes of these actions, evaluate them, and vary the approach when needed. Newell et al. (1962) called this process heuristic search through a problem space. In this view, switching between search strategies can account for the creative solution (Simon, 1986). The final step in the creative process, according to Amabile, is 4) response validation, which is similar to Walla’s verification phase, and involves evaluating possible responses against factual knowledge and other criteria, along with implementing and testing the idea (Amabile, 1983; Amabile, 1996).

Concerning the incubation phase, there is evidence that some insightful ideas arise when a complex problem is temporarily set aside. Whereas some authors associated this process with the ability to abandon unproductive search strategies, i.e., “productive forgetting” (Simon, 1966; Finke, 1996), others point to the role of defocused attention (Martindale, 1999; Sarathy, 2018).

In a line of work that focuses on the component cognitive operations (Sternberg, 1986a; Sternberg, 1986b; Sternberg, 1988), or “sub-processes” that compose complex cognition, the overall creative process was examined in more detail (Lubart, 2000). The first phase of the creative process (problem definition) includes selective encoding which is responsible for updating relevant and inhibiting irrelevant information (Benedek et al., 2014) and leads to problem representation in working memory. Selective comparison is responsible for 1) recalling relevant knowledge from long-term memory, and 2) mapping the relations between new and extant knowledge (Markman and Gentner, 1993). Selective comparison allows discovering a new relationship between new and already acquired information. Finally, novel solutions during the idea generation phase arise from the combination and recombination of knowledge in working memory (Sternberg, 1988). Mumford et al. (1991) have further addressed mechanisms of knowledge combination and proposed that reasoning, analogy use, and divergent thinking account for creative solutions. Sternberg (1986b) highlights also the role of meta-components in problem finding, problem definition (and redefinition), and strategy choice. Some theorists also refer to these processes as executive functioning (Miller and Cohen, 2001).

Finke et al. (1992) developed the Geneplore model of creative cognition and distinguished between generative and exploratory phases of creative search. The idea generative phase comprises strategies such as knowledge retrieval, synthesis, and categorical reduction (see Gilhooly et al., 2007 for the description). The generative phase results in the production of *preinventive structures*—preliminary models which are characterized by novelty and ambiguity. These characteristics of preinventive structures afford numerous possibilities for the selective combination of their properties during exploratory phase. Strategies that allow further exploration of these structures are, for example, searching for potential functions, attributes or limitations, hypothesis testing, and conceptual interpretation. As generation and exploration cycles repeat, the preinventive structures could be partially modified or completely replaced by the new ones.

Repetitions of Geneplore cycles and switching between generative and explorative strategies may be accompanied by changes in attentional focus. Indeed, there is evidence indicating that early stages of the creative process may involve instances of defocused attention, whereas later stages may require more focused attention (Dorfman et al., 2008; Kaufman 2011; Zabelina et al., 2016).

Martindale (1999) proposed that creative people are characterized by a better ability to shift between focused and defocused attention as a function of task demands. This claim has received empirical confirmation (Zabelina and Robinson, 2010). In terms of the Geneplore model, it means that the effective creative process may involve enhanced switching between generative and explorative strategies.

**FIGURE 2:**
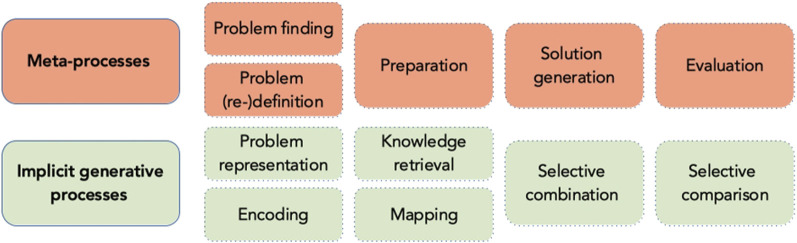
Two-level view of the creative process.

In summary, drawing on the work by Sternberg (1986a; 1986b; 1988; 2012), Amabile (1993;1996), Finke et al. (1992), Beghetto and Corazza (2019), we argue further that the creative process is a multistage dynamic process which builds on existing knowledge and is guided by a productive strategy search. This search is characterized by alternation between generative and explorative thinking. Importantly, generative and explorative cycles could unfold on two levels: a strategy could be discovered by explicitly reflecting on the task demands and previous problem-solving experience, i.e., at a meta-level, but it could also happen on the implicit level and be a result of trial and error search and exploration of associations between task, actions, and outcomes (Figure 2). This view is reminiscent of dual-process models (system 1, system 2) of human cognition (Crowley et al., 1997; Stanovich and West, 2000; Kahneman, 2011).

## Cognitive Components of the Process of Designing and Programming Robots

Drawing on principles of constructionism, Kolodner (2002) introduced a learning model that incorporates design and inquiry activities organized in two interrelated cycles: the “Investigate and Explore” cycle, where students acquire knowledge and generate ideas, and “Design/Redesign” cycle, where knowledge is applied. We can note that the model instantiates the basic principle of the Geneplore model of creative cognition (Ward et al., 1999), where the generative search alternates with explorative processes. Given the resemblance, it seems reasonable to apply existing models of creative cognition to analyze mental processes that underlie robotics activities.

The initial step in building and programming a robot is presenting the problem to be solved. For example, students are given a task to build a mobile robot and program its basic movements. This could be, for example, a creation of a robotic system that models a human heart (Cuperman and Verner, 2013), or programming a mail-delivery robot (Alves-Oliveira, 2020). A common feature of these robotic challenges is that they are poorly structured, have multiple solution paths, i.e., could be solved using different strategies, and do not have a single criterion for evaluating the solution.

From a cognitive point of view, the first step in the process of creating a robotic device is problem identification, in which a problem solver has to elaborate a problem representation. In terms of robotics, this implies analysis of the system's requirements and translation of these requirements into design specifications (Pahl and Beitz, 2007). In information processing terms, this step could be accomplished through selective encoding, i.e., selecting relevant elements of a problem and suppressing those that are not relevant for task completion (Sternberg, 1988; Benedek et al., 2014). Another important process is the retrieval of relevant information from long-term memory (Smith, 1995). Presumably, this is done via selective comparison (Sternberg, 1986a; 1986b), in which problem solver aligns existing knowledge and previous problem-solving experience with the characteristics of the new challenge (Holyoak, 1984; Mumford et al., 1991). It involves a comparison of critical elements such as goals, procedures, and constraints encountered in similar problems. In practical terms, with respect to generating ideas for a robot’s design, students spend time thinking about known solutions and how they might be reused in the new task (Kolodner, 1994). This process helps learners to identify the gaps in their existing knowledge. When the problem is new and procedural and dispositional knowledge is lacking, a great deal of learning takes place (Amabile, 1983). For example, in the study of Cuperman and Verner (2013), before building a robotic model of the human heart students had to carry out investigations to learn the principle of the heartbeat mechanism. If the domain-relevant skills and knowledge are sufficient to afford a range of possible pathways to explore, students immediately start the process of building a robot after the problem has been defined.

The process of solution generation in robotics problems is often paralleled with implementation, i.e., designing the robots. As robotics problems are often ill-defined, finding possible solutions for each design specification requires a search among numerous potential alternatives within a space of possibilities (Ball et al., 1997). There is evidence that generating few ideas at this stage leads to the restriction of the search space and poor designs, as students became “fixated” on concrete solutions too early (Fricke, 1996).

The generation stage in robotics design involves mental and physical synthesis of building components and creating functional prototypes. Functional prototypes of robots that result from initial generative processes may be viewed as preinventive structures (Finke et al., 1992) that are assessed for appropriateness and other criteria and are further modified during the exploratory phase*.* Evaluation of the prototypes naturally leads students back to the first stages of the creative process—redefining the design specifications, as well as gathering task-relevant information (Suwa et al., 1999). This iterative process of perceiving an emerging design and making a change to it allows to learn new affordances and often leads to unexpected discoveries (Schön and Wiggins 1992; Kelly and Gero, 2014).

The process of a robot’s design is followed by an iterative, trial-and-error phase of programming the robot’s moves, testing, and modifying its design and software code (Nemiro et al., 2017; Alves-Oliveira, 2020; Chevalier et al., 2020). In the later cycles of the process of creation of the robotic model, students move beyond a trial-and-error method and start developing their own heuristic approach, which allows them to come up with original technical solutions (Hayes, 1978; Altshuller, 1988; Sullivan and Lin, 2012; Sullivan, 2017).

Barak and Zadok (2009) described three explorative strategies that lead learners to inventive solutions in robotic tasks. The first strategy the authors called “assigning a new function”, where students find a new use for an already existing robot’s movement. The second strategy involves the elimination of a component from the system. This heuristic has been extensively described in TRIZ (Altshuller, 1988). The third strategy consists of examining physical objects available in the environment and trying to apply them to solve a problem. Sullivan (2011) called this last strategy “utilizing environmental affordances”. Attentional mechanisms, and more specifically, diffused attention, may be important for this strategy as it helps to notice some environmental cues leading to the generation of novel ideas (Sarathy, 2018; Zabelina, 2018).

Sullivan (2011) described the process of constructing a robotic model in terms of troubleshooting cycles and rapid prototyping rounds, in which students fluently move between 1) writing code, 2) testing the robot, 3) analyzing problems, 4) proposing changes to the model, and 5) testing the device again. The author’s detailed analysis of the solution trajectory shows that each troubleshooting round includes three key stages: 1) problem identification, 2) idea generation and strategy choice, and 3) reflections on the progression of the problem-solving process. Sullivan (2011) described a case of a robotics programming activity in which the solution process consisted of 17 troubleshooting cycles and was two-fold: first, an explorative strategy was used to discover novel affordances of materials and then the problem was redefined, i.e., meta-level reasoning was applied.

**FIGURE 3:**
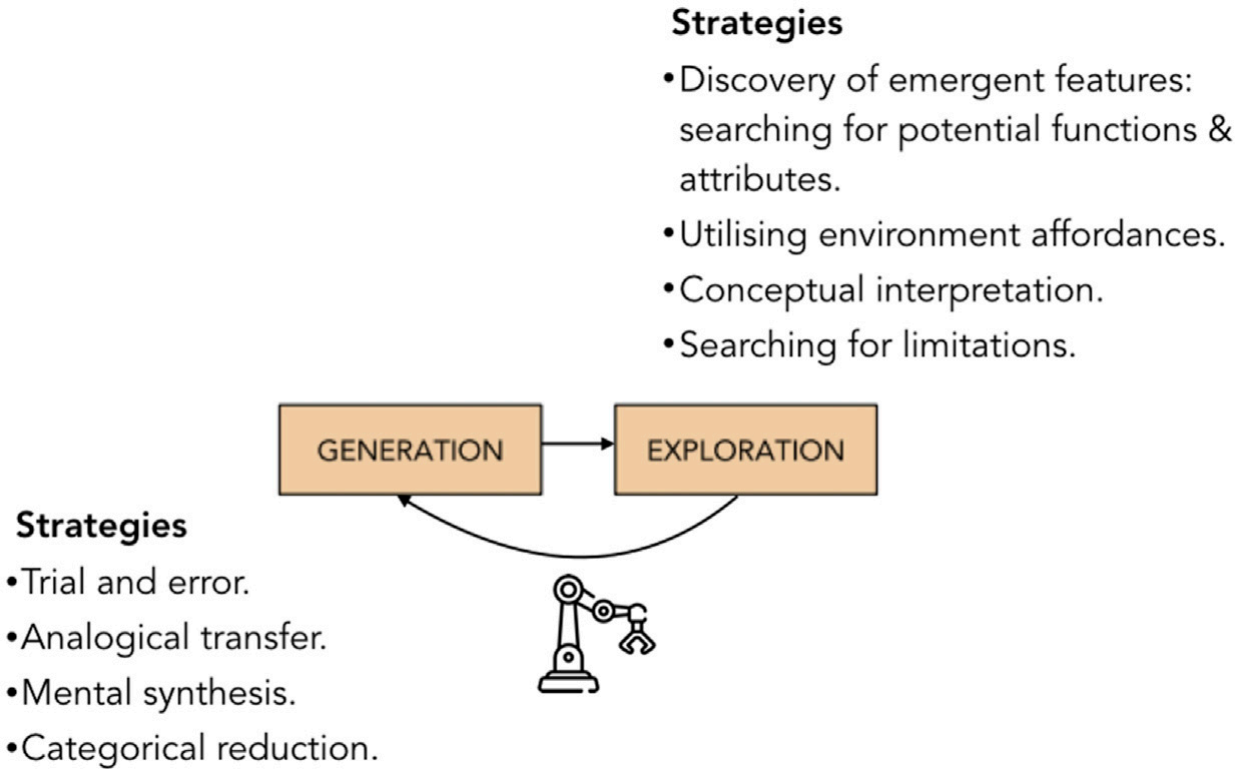
Solution generation and exploration.

To summarize, the process of building robotic models can be characterized by a constant search and movement back and forth between generative and explorative thinking (Figure 3). The creation of a robotic model involves using generative strategies, like memory retrieval (Sullivan, 2011), brainstorming (Nemiro et al., 2017), mental synthesis, and analogical transfer (Barak and Zadok, 2009; Cuperman and Verner, 2013), as well as explorative strategies–attribute finding, conceptual interpretation (Barak and Zadok, 2009; Chan and Schunn, 2015), and utilizing the environmental affordances (Sullivan, 2011). As our analysis suggests, the search for a solution in a robot construction process involves not only switching between generative and explorative strategies but also switching between levels of thinking at which these strategies operate. One may suppose that the practice of alternating between two different modes of cognition, generative and explorative, coupled with implicit and metacognitive processes that work in parallel, could result in better coordination between these components and promote student’s cognitive flexibility. Recent instructional models for teaching creativity via educational robotics also underscore the role of generative, explorative, and meta-components (Chevalier et al., 2020; Yang et al., 2020). Another possible explanation that can account for the promotion of student’s creative potential by robotics programs is that the process of engaging in collaborative construction of robotic devices leads not only to novel physical artifacts but also to the emergence of new mental tools–implicit and meta ideational strategies. Thus, engaging in physically, technologically and socially situated robotics problems could lead to the development of creative expertize in students.

This rather brief analysis does not aim to provide an exhaustive description of the process of robot building and programming. Rather, we aimed to illustrate that the solution trajectory in robotics problems could share parallels with the creative process and could be described in cognitive processing terms that are often cited in conceptions of creative cognition.

## Creative Processes in Autonomous Robots

In previous sections, we have described creativity as a socially and materially situated practice that unfolds over time through perceiving and exploring material and technological affordances and generating novel artifacts. In addition to student’s conative and cognitive factors, the confluence model of creativity emphasizes the role of the environment in translating the student’s creative potential into novel and useful products. Evaluating such models of human creativity is, however, challenging in natural settings due to ethical concerns and difficulties in isolating hypothesized variables.

Modern machine learning algorithms allow roboticists to develop autonomous agents able to learn by exploring their environment. Contrary to computational creativity, research in robotics using reinforcement learning is also situated, in the sense that it uses methods applicable for embodied agents. In this regard, the robot becomes a perfect tool to study and model the emergence of creativity.

Up to this point, we have used the term “robot” in a passive form and considered it as a tool to develop human creativity. In this section, we will change our perspective to consider the robot as a testbed to implement and verify our model of the creative process. Implementing a model for physical experimentation requires specifying all internal structures and processes involved (Fong et al., 2002).

Building on the description of processes outlined in the preceding sections, we argue that to be able to simulate the creative process, autonomous agents should be able to:

1. Acquire new knowledge and learn.
2. Reactivate and reuse knowledge in a wide range of environments.
3. Select and change problem-solving strategies.
4. Use meta-reasoning to define and redefine problems, evaluate the process and artifacts.

A collection of automatic processes capable of producing behavior that would be deemed creative in humans is called a “creative system” by Wiggins. The Creative Systems Framework (Wiggins, 2006) describes the creative system in terms of a search process that goes through a conceptual space to generate artifacts. This exploratory search is coupled with a metacognitive search process that operates within all possible conceptual spaces. Linkola et al. (2020) attempted to apply the notion of Wiggins exploratory search to learning agents. Drawing on concepts from Markov Decision Processes (MDPs), the Creative Action Selection Framework (Linkola et al., 2020) provides a formal account of the agent’s action choice based on the value, novelty, and validity of artifacts and concepts.

Several authors suggested that modern reinforcement learning algorithms based on MDPs could allow simulation of the creative process in autonomous agents (Vigorito and Barto, 2008; Schmidhuber, 2010; Colin et al., 2016). Reinforcement learning (RL) resembles the creative process as both involve interaction between a decision-making agent and its dynamic, uncertain environment, when the agent is searching for a solution to a given problem. In reinforcement learning problems, an agent explores the space of possible strategies and gets feedback based on the results of its decision making. This information is used to deduce an optimal policy (Kober et al., 2013). According to Colin et al. (2016), the agent’s policy changes within hierarchical reinforcement learning algorithms resemble the change in strategies that happens during creative processes.

One of the challenges of reinforcement learning is the dilemma between exploration and exploitation (Sutton and Barto, 1998). To obtain more reward, a reinforcement learning agent must choose actions that have been effective in the past. But to discover such actions and make better action selection in the future, the robot has to try actions that it has not selected before. The creative process is also marked by the constraint between new and already existing problem-solving strategies (Collins and Koechlin, 2012) and by the necessity to build upon previous experience and knowledge in order to extend or break with them to generate novelty.

One way to address this dilemma is to introduce intrinsic motivation in RL, i.e., modifying the reward function to improve the performance of an agent (Singh et al., 2010). Whereas the traditional approach to RL is to provide reward only in case of task achievement, intrinsically motivated agents are also encouraged by “cshaping” rewards for discovering novel, surprising patterns in the environment (Ng et al., 1999). According to Schmidhuber (2010), the discovery of these novel regularities in curiosity-driven exploration would be marked by an impressive reduction in computational resources.

Recent advances in reinforcement learning are associated with the introduction of deep reinforcement learning, showcasing agents learning to play games which have long been considered as very complex for artificial agents (Mnih et al., 2015; Silver et al., 2016; Schulman et al., 2017). One of the major limitations of RL algorithms is, however, their high computational cost to learn new environments. Although RL has been successfully used to autonomously solve complex tasks, learning to solve these tasks requires large time investments. This is due to the fact that in order to converge on a good solution, RL agents require a significant number of explorative interactions with the environment.

Several approaches have been introduced to reduce reinforcement learning time; these include learning through other agent’s advice in a shared environment (Saunders, 2012; Silva and Costa, 2019), and learning from human demonstrations (Argall et al., 2009; Fitzgerald et al., 2018). Another way to overcome the drawback of time-consuming exploration is to enable machine learning algorithms with the ability to transfer and reuse previously acquired knowledge across tasks using a case-based reasoning approach (CBR) (Riesbeck and Schank, 1989; Kolodner, 2014).

CBR begins with a problem representation of the situation in which the case can be used. Problem representation is compared with cases stored in a case base using specified similarity measures. If relevant cases exist, they are retrieved, adjusted, and reused in the problem at hand (Aamodt and Plaza, 1994; De Mantaras et al., 2005). Given that CBR has already been coupled with TRIZ problem-solving strategies and showed its potential to accelerate innovation design (Robles et al., 2009; Ching-Hung et al., 2019), its application to speed up RL seems promising.

Recent attempts to combine the advantages of reinforcement learning with case-based reasoning can be found in Glatt et al. (2020), Bianchi et al. (2018). Whereas Deep Case-Based Policy Inference algorithm accelerates learning by building a collection of policies and using it for a more effective exploration of a new task, the latter, Transfer Learning Heuristically Accelerated Reinforcement Learning algorithms (TLHARL), speeds up the RL process using CBR and heuristics. Bianchi et al. (2018) have shown that TLHARL improved significantly the learning rate in two domains – robot soccer and humanoid-robot stability learning.

The success of a system using CBR techniques depends on the ability of the system to retrieve, redefine, and reuse cases. To detect reasoning failures, improve the similarity assessment measure and the case adaptation mechanisms of the CBR system, meta-reasoning techniques are used. Arcos et al. (2011) have described an introspective reasoning model enabling a CBR system to learn autonomously to improve multiple facets of its reasoning process. The model performs five distinct functions: 1) monitoring the CBR process; 2) assessing the quality of proposed solutions; 3) identifying reasoning failures; 4) proposing goals; and 5) evaluating the impact of proposed improvements. Enabled with meta-reasoning, the system can identify and repair the sources of failures and thus incrementally adapt to the new problem situation.

CBR systems have their limits as well, however. Whereas they are effective when dealing with cases that bear resemblance to the task that has already been experienced by the robot, CBR systems have limited efficiency when they encounter novel problems. Parashar et al. (2018) have introduced an architecture enabling an agent to cope with novelty. The work addresses the issue raised by Sarathy and Scheutz (2018), Konidaris et al. (2018) and combines planning and reinforcement learning approaches. This combination of top-down and bottom-up approaches makes the work of Parashar et al. (2018) especially relevant for the context of creative problem solving in robotics. The authors proposed a three-layered agent architecture, with 1) object-level reasoning acts based on the information encoded from the environment; 2) deliberative reasoning, responsible for plan construction and action based on object-level information, and 3) a meta-reasoning layer responsible for problem construction and re-construction based on object-level and deliberative-level information and learning history. Meta-level reasoning also allows to control switching between object-level and deliberative strategies.

**FIGURE 4:**
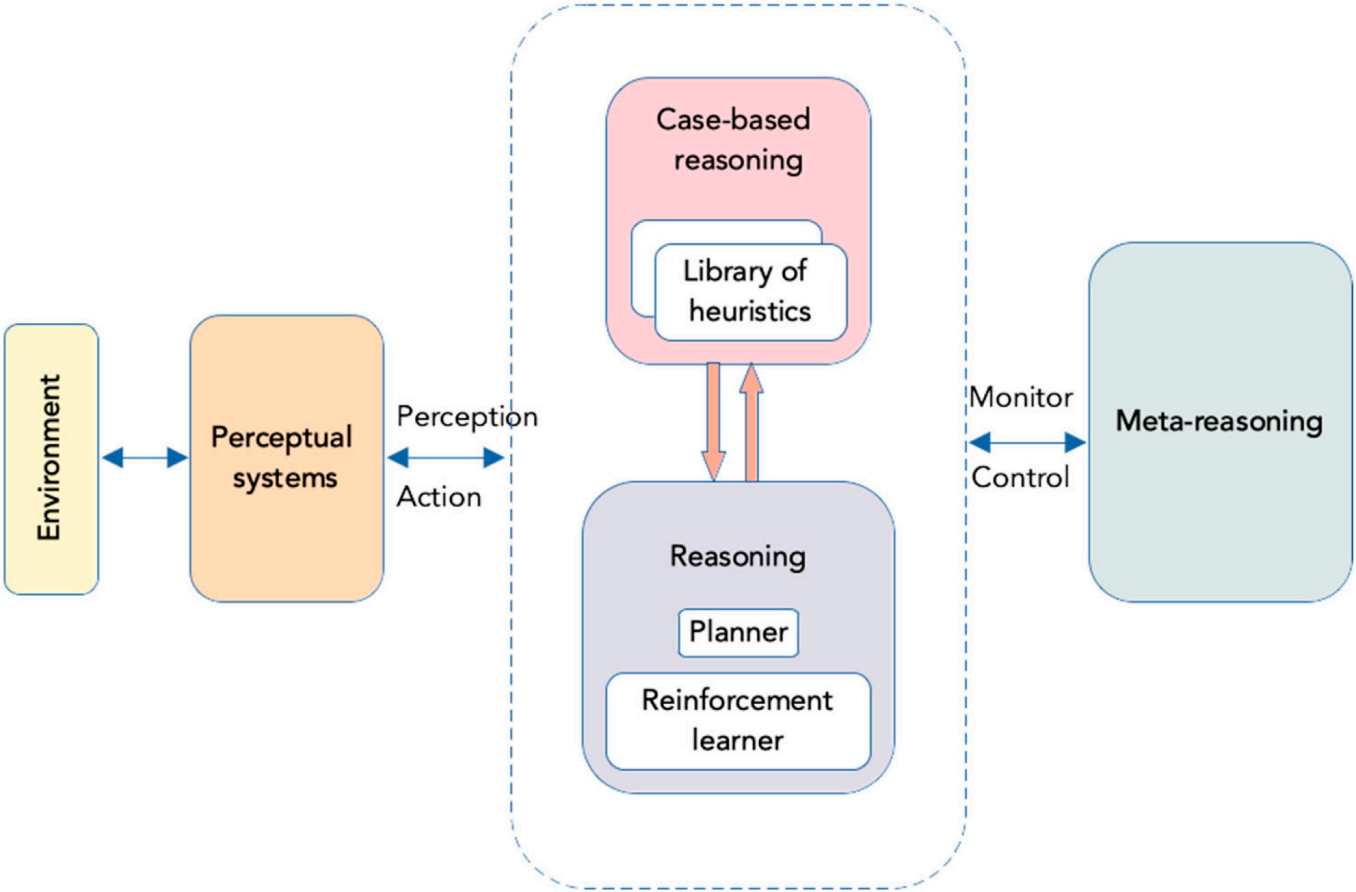
System architecture.

In this section, we have outlined the techniques that could be a possible starting point for modeling the creative process in artificial systems. A tentative model of system architecture is shown in Figure 4. A combination of these or similar techniques (Augello et al., 2018; Edmonds et al., 2020; Goel et al., 2020) might result in a hybrid approach for design agents capable of addressing novelty and handling MacGyver-type problems using affordances (Sarathy and Scheutz, 2018).

## Discussion

We began with the observation that whereas numerous studies have shown a positive effect of constructing and programming robots on creativity, little attention has been paid to the mechanisms that can account for this effect. Educational robotics has been considered as an inherently creative activity. To address this gap, we have examined the process of designing and programming robots with respect to existing models of creative cognition. Our analysis resulted in a description of the creative process as a multistage process, which builds on existing knowledge and involves trial-and-error, generative, explorative, and metacognitive components. Next, we reviewed some recent techniques enabling robots to simulate the creative process and proposed that a combination of reinforcement learning, case-based reasoning, and meta-reasoning methods has the potential to design robots that can address novelty and solve MacGyver-type problems.

Many questions remain, however. First, as the confluence model (Lubart et al., 2015) specifies, a combination of cognitive mechanisms is a necessary condition for the creative product to appear. Conative and environmental aspects must also join to engage creative work. And yet, what is even more striking, our current understanding of human creativity is far from complete, as psychologists still do not know precisely how these multiple factors interactively work together to influence creative production. For example, what is the optimal level of a person’s intrinsic motivation and tolerance to ambiguity to achieve a creative outcome? Does intrinsic motivation enhance the use of certain strategies? How do contextual variables, such as resources or an uncooperative environment, modify the creative process? Is there a threshold for the various creativity predictors, under which creativity cannot arise? Can creativity occur if one cognitive or conative feature is completely missing?

In the case of robotics, even though certain cognitive processes have been emulated, it is still not clear how robots construct problem representations, what is the nature of these representations, or whether robots can autonomously find problems to solve. Regarding the non-cognitive aspects of Lubart et al.’s confluence model (2015), the question arises as to which extent robots can be designed to incorporate conative aspects.

In the light of conceiving robots that should act as social agents, their potential “personality” moves into the spotlight. If the genetic contribution to personality is lower than to cognition (Loehlin and Nichols, 2012), it should theoretically be easier to program robots that develop a certain “personality”, and this is what some researchers have tried to do (Goetz and Kiesler, 2002; Lee et al., 2006; Woods et al., 2007; Tapus et al., 2008), notably regarding the introversion/extraversion trait (Goetz and Kiesler, 2002; Lee et al., 2006; Tapus et al., 2008). The important question is to which extent robots can imitate the major creativity-related traits, including perseverance, tolerance of ambiguity, openness to new experiences, and risk-taking (Lubart et al., 2015). Regarding openness to new experiences, which is viewed as the most relevant personality trait for creativity (McCrae, 1987; Feist, 1998; Feist, 1999), no direct attempts have been realized to program an “open-minded” robot. Agnoli et al. (2015) found that attentional processing of apparently irrelevant information (irrelevance processing) acts as a moderator between openness and creative performance. It is imaginable that robots could be programmed for irrelevance processing and, as such, embody a certain “openness”.

With respect to tolerance of ambiguity, creative performance is favored by encouraging people not to be satisfied by hasty, partial, or non-optimal solutions to complex problems (Lubart et al., 2015). Re-interpreted as a metacognitive skill, ambiguity tolerance refers to the “ability to cope with increasing sensitization to novel features of a phenomenon in order to redefine prior conceptual interpretations, contingent on trust and motivation” (Lakhana, 2012, p. III). When defined in this way, it is imaginable that robots could be programmed to display ambiguity tolerance.

As far as motivation is concerned, most attention has focused on intrinsic motivation as a positive condition for creative engagement and achievement in humans (Collins and Amabile, 1999). As described in the previous section, there are currently attempts to create intrinsically motivated robots using the reinforcement learning approach, especially regarding their intrinsically motivated open-ended learning (Schmidhuber, 2010; Santucci et al., 2020). The research is also marked with some encouraging attempts (Parisi and Petrosino, 2010; Kashani et al., 2012; Daglarli, 2020) to simulate robot’s emotional states.

When it comes to the environmental aspects fostering creative performance, as we have mentioned in the previous section, there are already robots that cooperate and transfer knowledge (Silva and Costa, 2019). Projects like the Curious Whispers (Saunders et al., 2010), which study the potential of artificial society’s evolution within a human physical, social, and cultural environment, are being investigated.

The possibility of comparing humans and robots in terms of creativity has traditionally focused on the productions of both, looking at whether humans and robots may produce similar or different creative work. Questions concerning the relative originality or productivity of humans and computers are raised. In contrast, our focus has been process-oriented. Do humans, who engage in a robot construction project, involve specific types of cognition that foster the development of creativity? Do robots, which instantiate artificial intelligence algorithms, engage in creative processing as humans do spontaneously? A robot may best be compared with a human baby who is learning and making discoveries by exploring the environment. As Smith and Gasser (2005), p.13 argued, “starting as a baby grounded in a physical, social, and linguistic world is crucial to the development of the flexible and inventive intelligence that characterizes humankind.” We suggest that full-fledged creativity is in a robot’s “zone of proximal development” (Vygotsky, 1967): what a robot cannot reach alone, it may reach with the help of a human teacher. As we have seen, robots, even in their simplest form, could also aid humans in their creative endeavors. Hence, humans and robots could fruitfully complement one another in the elaboration of creative outcomes.

## Conclusion

In this work, we have described the creative process in information and cognitive processing terms, suggesting that computer science and cognitive psychology have had a mutual impact on each other. This influence has led to the development of a common language among psychologists and computer science engineers. As our analysis suggests, creativity research in psychology has accumulated a large set of empirical data and theoretical knowledge on human creativity, which can be useful for both an analysis of the benefits of robot design and programming for students to develop their own creativity, as well as the design of artificial agents, robots, who are themselves capable of being creative. After providing models of human creativity for machine design, psychology could gain new insights from the implementation and verification of these models in embodied agents. Interdisciplinary dialogue and collaboration between psychologists and roboticists could contribute toward better understanding of creativity and the future development of both creative humans and creative robots.

## Data Availability

The original contributions presented in the study are included in the article/Supplementary Material, further inquiries can be directed to the corresponding author.

## Footnotes

1 In this article, the term “creative” refers to a response that is: adapted to the problem situation and has not been taught in class (children), adapted to the problem situation and has not been previously programmed for (robot).

## Abbreviations

CBR, case-based reasoning; ER, educational robotics; FC, figural creativity; MDPs, markov decision processes; RL, reinforcement learning; TCT-DP, test for creative thinking-drawing production; TLHARL, transfer learning heuristically accelerated reinforcement learning; TTCT, torrance test of creative thinking.
